# Bioactive compounds and antioxidant activities of *Quercus salicina* Blume extract

**DOI:** 10.1007/s10068-020-00755-1

**Published:** 2020-07-23

**Authors:** Thinzar Aung, Marie Anna Dominique Bibat, Chang-Cheng Zhao, Jong-Bang Eun

**Affiliations:** Department of Integrative Food, Bioscience and Biotechnology, Graduate School of Chonnam National University, Gwangju, 61186 South Korea

**Keywords:** Phenolic, Flavonoid, Antioxidant, Extract, Oak

## Abstract

*Quercus salicina* Blume (QS) is an oak species that is indigenous to Japan and Korea. Historically, extracts of leaves, stems, barks and buds from the QS tree had been extensively utilized as herbal medicines. As rich sources of natural antioxidants, QS extracts could prevent oxidative stress and the occurrence of related neurodegenerative diseases. In pharmaceutical applications, decoction or infusion of comminuted QS powder is prepared as an herbal tea for oral use. Various extraction methods and extracting mediums showed the potential antioxidant activities of QS extracts, as well as the different types and levels of bioactive compounds found in them. Due to their functional properties and possibly low-level of cytotoxicity, the potential application of QS extracts as a novel food ingredient could be considered. In this review paper, a brief overview about QS extracts and their bioactive components, antioxidant activities, toxicity and technological applications is described based on previous works.

## Introduction

Since a thousand years ago, *Quercus* (oak) trees have been considered valuable by mankind. From acorns as food, charcoal for metal melting or wood for construction and furniture making, the basic resources from oaks are numerous to the extent that certain areas particularly regard them as “tree of life”. Comprising more than 400 species distributed worldwide, *Quercus L.* belongs to the *Fagaceae* family and is the most diverse northern temperate tree genus. Oaks can survive in contrasting phytoclimates, from temperate and subtropical forests to Mediterranean evergreen woodlands. Specifically, they have a wide geographical range, from subalpine to semiarid forests, wetlands and even reaching the Tropics (Gil-Pelegrín et al., 2017).

Aside from their obvious functions as timber and food source, the species of *Quercus* are also utilized because of their medicinal properties. For instance, the bark, leaves and fruit of these trees have been used in traditional medicine either as topical treatment for burns and hemorrhage, or as oral treatment to cure gastrointestinal diseases such as dysentery (Bahador & Baserisalehi, 2011; Deryabin & Tolmacheva, 2015). They have also been utilized to treat diarrhea, dermatitis and throat infections (Berahou et al., 2007; Kim et al., 2008). Accordingly, many scientists have shown interest in the study of this species in recent years and have resulted to discoveries that describe their chemical constituents, functional properties and beneficial effects. Studies have correlated the therapeutic effects of *Quercus* extracts with their antimicrobial properties and have presented the potential of maximizing these properties to produce more natural remedies which can solve the problem of antibiotic resistance (Bahador & Baserisalehi, 2011). Furthermore, numerous works have reported the detection of high quantities of phenolic compounds and antioxidant activities in *Quercus* extracts.

*Quercus salicina* Blume (QS) is one of the many species of *Quercus* that is native to Japan and southern parts of South Korea. It is known for its anti-inflammatory, antiedemic, litholytic, diuretic and antibacterial properties (Kim et al., 2008; Kim et al., 2012). Moriyama et al. (2007; 2009) and Heo et al. (2012) researched about Urocalun^®^, a commercially available herbal medicine with *Q. salicina* Blume and *Q. stenophylla* Makino extracts. They investigated the mechanisms involved in its function of treating urolithiasis, and the findings of their studies suggested that the antioxidative activity of the extract plays a major role in preventing stone formation and recurrence of urolithiasis. Gu et al. (2014) exploited the antibacterial property of QS by utilizing its extract in cream. Their study also exhibited that QS extracts have inhibitory effects on the growth of *S. aureus*, *B. subtilis*, *P. acnes* and *P. aeruginosa*. Extracts from QS leaf, bark and duramen were also found to have anti-allergic activities (Bak et al., 2011). On the other hand, Cho et al. (2011) validated the anti-inflammatory properties of the methanolic extract of QS and its potential use in treating macrophage-mediated inflammatory diseases.

However, most of the researches on QS delved into the bioactive compounds and antioxidant activities of its extract. Studies have revealed that the extracts from the leaves and bark of QS contain a large concentration of phenolic compounds and were found to be significantly higher than two other *Quercus* species and *Rhaphiolepis indica*. In this regard, it has been suggested that QS is a promising natural ingredient that can be utilized in the pharmaceutical setting (Kim et al., 2016; Tuyen et al., 2016). Moreover, Lee and Park (2011) demonstrated the strong antioxidant activity and inhibitory effect of QS extract on tyrosinase and elastase, and they recommended the application of this extract in new functional cosmetics. While current studies mostly discussed its applicability in the pharmaceutical and cosmetic industries, the presence of a considerable amount of bioactive components in QS extracts could serve as basis to propose its use as an added-value ingredient in the food and nutraceutical industry. Apart from its existing application as herbal tea in Korea and Japan, with the latter having records on its consumption dating back to 1969 (Bone & Mills, 2013), the limited utilization of QS extracts could be further expanded through product development studies.

The present review, which highlights the currently published information related with the phytochemical and biological activities, as well as the toxicity and industrial applications of QS extracts, aims to be a useful resource for validating the future application of QS as a functional food ingredient or as a raw material for related industries. On a side note, this review is limited to English and Korean language publications.

## Bioactive components in *Quercus salicina* Blume extracts

Phenolic compounds comprise a diverse group of molecules classified as secondary metabolites in vegetables, fruits and plants that have a large range of structures and functions. Generally, they can be classified into water-soluble compounds including phenolic acids, phenylpropanoids, flavonoids and quinones, and water-insoluble compounds such as condensed tannins, lignins and cell-wall bound hydroxycinammic acids (Haminiuk et al., 2012). It has been reported that phenolic acids and flavonoids are the major class of phenolic compounds (Cai et al., 2004). In phenolic compounds, phenolic acids consist of two subgroups, namely, hydroxybenzoic and hydroxycinnamic acids (Bravo, 1998). Flavonoids constitute the largest group of plant phenols and naturally generating phenolic compounds (Harborne et al., 1999) and have the basic skeleton of diphenylpropanes. Table 1 shows the individual phenolic and flavonoid compounds identified in different parts of the QS plant based on various studies. Extraction was performed using different solvents, while chromatographic techniques such as high-performance liquid chromatography (HPLC) and thin layer chromatography (TLC) had been utilized to identify and quantify specific phenolic compounds in QS extracts.

**Table 1:** Phenolic and flavonoid compounds detected in the different plant parts of *Quercus salicina* Blume species

Part of the plant	Origin	Extracting medium	Name of phenolic and flavonoid compounds detected	Amount	Method of detection	References
Leaves	Korea (southern coast and island)	Acetonitrile, hydrochloric acid and ethanol	Phenolic acids	(µg/g)	HPLC with PDA detector Separation on YMC-Pack ODS-AM-303 (5 M, 250 mm × 4.6 mm I.D)	Kim et al. (2012)
			Gallic acid	47.57		
			Pyrogallol	3708.30		
			Homogentisic acid	856.48		
			Protocatechuic acid	1199.60		
			Gentisic acid	9587.50		
			Chlorogenic acid	6081.11		
			ρ-Hydroxybenzoic acid	317.92		
			Vanillic acid	197.04		
			Syringic acid	130.02		
			Caffeic acid	394.50		
			Vanillin	12.06		
			*ρ*-Coumaric acid	162.81		
			Ferulic acid	297.11		
			m-Coumaric acid	11.86		
			Salicylic acid	553.11		
			*ο*-Coumaric acid	16.08		
			Resveratrol	35.27		
			*t*-Cinnamic acid	3.84		
			Veratric acid	166.31		
			*β*-Resorcylic acid	403.26		
			Flavonoid	(µg/g)		
			Rutin	167.4		
			Quercetin	26.00		
			Naringenin	7.05		
			Hesperetin	5.05		
			Formononetin	2.37		
			Biochanin A	14.52		
			Naringin	1023.00		
			Kaempferol	11.63		
			Myricetin	199.56		
			Hesperidin	63.85		
Leaves	NS	Ethyl acetate fraction	Quercitrin Catechin Kampferol Quercetin Isoquercetrin Hyperoside	NS	HPLC, TLC	Lee and Park (2011)
Leaves	NS	Aglycone fraction	Kampferol Quercetin	NS	HPLC, TLC	Lee and Park (2011)
Leaves	Ashiu Forest Research Station, Kyoto University, Japan	Ethanol	Free Phenolic acids	(mg/g DW)	HPLC (254 nm) UV detector Column Jasco RPC 18 (250 mm × 4.6 mm × 5 µm)	Tuyen et al. (2016)
			Benzoic acid	2.87		
			Ellagic acid	0.37		
Leaves	Ashiu Forest Research Station, Kyoto University, Japan	Ethyl acetate	Bound Phenolic acids	(mg/g DW)	HPLC (254 nm) UV detector Column Jasco RPC 18 (250 mm × 4.6 mm × 5 µm)	Tuyen et al. (2016)
			Gallic acid	0.12		
			Protocatechuic acid	0.15		
			Chlorogenic acid	0.93		
			*p*-Hydroxybenzoic acid	0.11		
			Vanillic acid	0.72		
			Ferulic acid	0.59		
			*p*-coumaric acid	0.54		
			Ellagic acid	1.1		
Barks	Ashiu Forest Research Station, Kyoto University, Japan	Ethanol	Free Phenolic acids	(mg/g DW)	HPLC (254 nm) UV detector Column Jasco RPC 18 (250 mm × 4.6 mm × 5 µm)	Tuyen et al. (2016)
			Chlorogenic acid	1.55		
			*p*-Hydroxybenzoic acid	0.25		
			Ellagic acid	1.83		
Barks	Ashiu Forest Research Station, Kyoto University, Japan	Ethyl acetate	Bound Phenolic acids	(mg/g DW)	HPLC (254 nm) UV detector Column Jasco RPC 18 (250 mm × 4.6 mm × 5 µm)	Tuyen et al. (2016)
			Gallic acid	0.03		
			Chlorogenic acid	0.32		
			Vanillin	0.16		
			*p*-coumaric acid	0.11		
Shoots	Korea (southern coast and island)	Acetonitrile, hydrochloric acid and ethanol	Phenolic acids	(µg/g)	HPLC with PDA detector Separation on YMC-Pack ODS-AM-303 (5 M, 250 mm × 4.6 mm I.D)	Kim et al. (2012)
			Gallic acid	128.31		
			Pyrogallol	3006.20		
			Homogentisic acid	1435.63		
			Protocatechuic acid	541.50		
			Gentisic acid	4452.30		
			Chlorogenic acid	3401.00		
			*ρ*-Hydroxybenzoic acid	247.00		
			Syringic acid	69.56		
			Caffeic acid	587.53		
			Vanillin	13.58		
			*ρ*-Coumaric acid	39.68		
			Ferulic acid	39.38		
			Salicylic acid	137.18		
			*ο*-Coumaric acid	22.00		
			Resveratrol	6.55		
			*t*-Cinnamic acid	1.87		
			Veratric acid	55.09		
			β-Resorcylic acid	279.64		
			Flavonoid	(µg/g)		
			(+) Catechin	576.43		
			Rutin	397.20		
			Quercetin	312.30		
			Naringenin	6.08		
			Hesperetin	5.02		
			Formononetin	8.20		
			Biochanin A	11.33		
			Naringin	261.30		
			Kaempferol	11.16		
			Myricetin	308.09		
			Hesperidin	100.83		

*HPLC* High Performance Liquid Chromatography, *TLC* Thin Layer Chromatography, *PDA* Photodiode Array detector, *NS* not specified, *DW* dry weight

External factors such as seasonal changes and different stages of maturity have generally been reported to contribute to the variations in phenolic and flavonoid compounds in *Quercus* species (Brossa et al., 2009; Makkar et al., 1991), but most studies regarding QS extracts investigated more on the differences in phenolic and flavonoid compounds composition based on the different parts of the QS plant or in comparison with other *Quercus* species. In terms of the parts of the plant, the leaves of QS have been shown to have higher contents of phenolic and flavonoid compounds compared with its shoot and bark. Tuyen et al. (2016) showed the significant differences between QS leaf and bark through their study in which they measured the free, bound and total phenolic and flavonoid contents of the extracts. They reported that the leaf and bark have a total phenolic content of 46.30 mg and 35.89 mg gallic acid equivalent (GAE) per dry weight of samples, while the total flavonoid contents were reported to be 24.72 mg and 1.41 mg rutin equivalent (RE) per dry weight of samples respectively. On the other hand, Kim et al. (2012) compared the total phenolic contents between the QS leaf and shoot and have derived a total amount of 25,702.13 and 16,461.82 μg/g, respectively. Proportional compositions of phenolic acids and flavonoids between the leaf and shoot were also contrasted, and the said study had shown that a higher percentage of phenolic acids can be found in the leaf (94.08%) compared to the shoot (87.86%) and vice versa for the flavonoid content proportion (5.92% in leaf; 12.14% in shoot). Particularly, vanillic acid and *m*-coumaric acid were only detected in leaves, while (+)-catechin (576.43 μg/g) was only detected in the QS shoot.

In addition, these two groups of researchers simultaneously compared the bioactive compounds composition of QS with other *Quercus* species, and both have concluded that QS contained the highest concentration levels of phenols and flavonoids. Kim et al. (2012) investigated the phenolic profile in leaves of five different *Quercus* species including *Q. salicina* Blume, *Q. acuta* Thunberg, *Q. phillyraeoides* A. Gray, *Q. glauca* Thunberg and *Q. myrsinaefolia* Blume and confirmed the presence of many chemical constituents. Among the individual phenolic compounds, gentisic acid was the highest, especially in leaves (9587.50 μg/g). Moreover, QS extracts had higher chlorogenic acid (6081.11 μg/g) and pyrogallol (3708.30 μg/g) content than other substances. The total flavonoid content of QS was also found to be the highest in contrast with the other *Quercus* species. Likewise, Tuyen et al. (2016) demonstrated the difference in bioactive compounds composition of QS with those of two other *Quercus* species namely *Q. serrata* and *Q. crispula*. Both leaf and bark extracts of QS were found to have the highest total phenolic compounds compared to other extracts.

## Antioxidant activities of *Quercus salicina* Blume extracts

Oxidative mechanism is related with the production of free radicals and reactive oxygen species (ROS) that can cause oxidative stress. To defend the organism against the pathologies associated with the attack of free radicals, antioxidants can be used as the protector (Antolovich et al., 2002). Antioxidants are essential to prevent degenerative diseases caused by oxidative stress, such as cancer, diabetes mellitus, rheumatoid arthritis, Parkinson’s disease and Alzheimer’s disease. Antioxidants can be classified into two types based on their solubility and line of defense (Panchawat et al., 2010). Based on the defense mechanism of antioxidants, there are preventive antioxidants such as superoxide dismutase (SOD), catalase (CAT), glutathione peroxidase (GTx) etc., radical scavenging antioxidants such as glutathione, vitamin C, uric acid, albumin, bilirubin, vitamin E, carotenoids, flavonoid etc. and repair and *de*-*novo* enzymes (Panchawat et al., 2010).

This article indicates the potential antioxidant activities of QS examined through 1,1-diphenyl-2-picrylhydrazyl (DPPH), 2,2′-azinobis (3-ethylbenzothizoline-6-sulfonic acid) diammonium salt (ABTS) free radical, reducing power, β-carotene bleaching method, ferric reducing antioxidant power (FRAP), hydroxyl radical scavenging activity, thiobarbituric acid reactive substances assay (TBARS), and the analysis of preventive antioxidants such as superoxide dismutase (SOD), catalase (CAT) and glutathione peroxidase (GSH-px). Different parts of the QS plant were extracted using different extracting mediums and studied their bioactive compounds and antioxidant activities (Table 2). Tuyen et al. (2016) reported that most of the *Quercus* species had higher antioxidant activities and QS species exhibited stronger activities than other *Quercus* species. They extracted free and bound phenolics from leaves and barks of three *Quercus* species using ethanol and ethyl acetate, then they analyzed the antioxidant properties using DPPH and ABTS radical scavenging activities, reducing power and β-carotene bleaching assays. From their results, free extracts from the barks of QS species showed the maximum inhibition activities in ABTS radical scavenging assays and free extracts from leaves and barks of this species also exhibited the strongest potential to inhibit β-carotene oxidation. However, for the DPPH scavenging activity, QS showed a similar trend with *Q. serrata* species. Another study also described that the extract from leaves of QS species showed higher DPPH scavenging activity than quercetin which was used as the control group (Kim et al., 2016). The particle sizes of QS leaf powder can also influence their antioxidant activities and bioactive compounds (Hong, 2019). In addition, QS leaf powder was extracted using ethanol and made the ethyl acetate and aglycone fraction. Among these fractions, the aglycone fraction showed the highest antioxidative effect (Lee and Park, 2011).

**Table 2:** Studies on the antioxidant activities of the different parts and different extracts of *Quercus salicina* Blume species

Part of plant	Type of study	Extraction type	Method for evaluation of antioxidant activity	Results	References
Leaves	Antioxidant capacity and phenolic contents analysis	Ethanol (99.5%)	DPPH	Free—0.067 (IC_50_ mg/ml)	Tuyen et al. (2016)
				Bound—0.079 (IC_50_ mg/ml)	
			ABTS	Free—0.523 (IC_50_ mg/ml)	
				Bound—0.559 (IC_50_ mg/ml)	
			Reducing power	Free—0.371 (IC50 mg/ml)	
				Bound—0.557 (IC_50_ mg/ml)	
			β-Carotene/linoleic acid	Free—96.15 (LPI %)	
				Bound—64.31 (LPI %)	
Leaves	Superfine grinding	Methanol	DPPH	Free—from 1.16 to 1.92 (mM TE/100 g DW)	Hong (2019)
			ABTS	Free—from 678.7 to 688.35 (mM TE/100 g DW)	
				Bound—from 733.39 to 740.84 (mM TE/100 g DW)	
			FRAP	Free—from 12.99 to 19.9 (mM TE/100 g DW)	
				Bound—from 15.93 to 19.31 (mM TE/100 g DW)	
			Hydroxyl radical-scavenging activity	Free—from 45.61 to 59.9%	
				Bound—from 43.7 to 60.38%	
Leaves	Antioxidative and cytotoxic effect	Ethanol	DPPH	6.63–92.41% Inhibition (Dose-dependent antioxidative effect)	Kim et al. (2016)
Leaves	Cytoprotective effect	Hot water	CAT	From 1.25 to 2.04 (U/mg protein)	Song et al. (2013)
			SOD	From 7.25 to 11.6 (U/mg protein)	
			GSH-px	From 3.19 to 4.85 (U/mg protein)	
Leaves	Antioxidative effect and active component analysis	Ethanol fraction	DPPH	18.21 (FSC_50_µg/ml)	Lee and Park (2011)
			ROS	0.87 (OSC_50_µg/ml)	
		Aglycone fraction	DPPH	8.25 (FSC_50_µg/ml)	
			ROS	0.039 (OSC_50_µg/ml)	
		Ethyl acetate fraction	DPPH	9.28 (FSC_50_ µg/ml)	
			ROS	0.054 (OSC_50_ µg/ml)	
Stems	Antioxidative compounds analysis	Methanol (30%)	DPPH	5.87 (IC_50_ µg/ml)	Kim et al. (2008)
			TBARS	2.15 (IC_50_ µg/ml)	
		Methanol (60%)	DPPH	11.01 (IC_50_ µg/ml)	
			TBARS	1.92 (IC_50_ µg/ml)	
		Methanol (100%)	DPPH	6.32 (IC_50_ µg/ml)	
			TBARS	2.38 (IC_50_ µg/ml)	
		Water	DPPH	6.21 (IC_50_ µg/ml)	
			TBARS	1.71 (IC_50_ µg/ml)	
		Chloroform	DPPH	37.5 (IC_50_ µg/ml)	
			TBARS	2.91 (IC_50_ µg/ml)	
Bark, buds	Antioxidant capacity and phenolic contents analysis	Ethanol (99.5%)	DPPH	Free—0.031 (IC_50_ mg/ml)	Tuyen et al. (2016)
				(IC_50_ mg/ml)	
			ABTS	Free—0.287 (IC_50_ mg/ml)	
				Bound—1.565 (IC_50_ mg/ml)	
			Reducing power	Free—0.271 (IC_50_ mg/ml)	
				Bound—1.223 (IC_50_ mg/ml)	
			β-Carotene/linoleic acid	Free—80.43 (LPI %)	
				Bound—77.26 (LPI %)	

*DPPH* 1,1-diphenyl-2-picrylhydrazyl free radical scavenging activity, *ABTS* 2,2′-azinobis (3-ethylbenzothiazoline-6-sulfonic acid) diammonium salt free radical scavenging activity, *FRAP* ferric reducing antioxidant power, *TBARS* thiobarbituric acid reactive substances assay, *SOD* superoxide dismutase activity, *CAT* catalase activity, *GSH-px* glutathione peroxidase, *LPI* Lipid peroxidation inhibition, *ROS* reactive oxygen species, *FSC50* free radical scavenging activity, *OSC50* active oxygen scavenging activity, *TE* Trolox equivalent

For the treatment of urolithiasis, the extract of leaves and branches from QS species prevented the oxalate-induced cell injury by its radical scavenging effect and suppressed the reduction of NADPH oxidase (Moriyama et al., 2007). Song et al. (2013) described that hot-water extract from the leaves of QS increased the activity of endogenous antioxidants, SOD, CAT, GSH-px in the alloxan-treated HIT-T15 cell. Therefore, they concluded that hot-water extract of QS had protective activity against alloxan-induced cell death in HIT-T15 hamster insulin-secreting cells. From these recent studies, it could be inferred that the relatively high antioxidative capacity of QS is strongly associated with its medicinal properties. Accordingly, extracts from various parts of QS plant have been widely applied in traditional folk medicine in Korea, China and Japan.

## Safety issue of *Quercus salicina* Blume extracts

Just as with other new food ingredients, availability of evidence to support the safety of use of QS as food is highly necessary for it to be recommended for food applications. Although current studies on QS did not directly probe into its toxicological properties, auxiliary experiments in some of these researches involved in vivo and cell viability assays which assessed the cytotoxicity of QS extracts. Assays that measured the mitochondrial reduction of 3-(4,5-dimethyl-2-thiazol)-2,5-diphenyltetrazolium bromide (MTT) on cells by QS extracts were performed in various works. Findings on the non-cytotoxic effects of hot water extracts of QS leaves (2.5–50 µg/ml) on HIT-T15 pancreatic β cells, methanolic extracts of QS leaves (62.5–250 µg/ml) on peritoneal macrophages and aqueous extracts from the combination of QS and *Q. stenophylla* Makino leaves and branches (3–30 µg/ml) on renal tubular epithelial (NRK-52E) cells have been presented by Cho et al. (2011) and Moriyama et al. (2007) and Song et al. (2013), respectively. One study reported that the ethanolic extract of QS leaves showed toxicity on 3T3-L1 preadipocyte cells at more than 100 μg/ml concentration and on human epithelial (A549) cells at over 200 μg/ml concentration (Kim et al., 2018). Additionally, Heo et al. (2012) performed in vivo testing of QS extract at a maximum dosage of 2000 mg/kg within a 14-day testing period, and they have reported no abnormal findings and zero mortality of tested mice.

Previous studies on the different *Quercus* species have suggested that certain parts of oak trees seem to present some level of toxicity to various terrestrial animals (Vinha et al., 2016). Historically, hydrolysable tannins and gallotannins have been associated with oak toxicosis, and immature leaves of oak are known to have the highest concentration of hydrolysable tannins (Martinson et al., 2007). However, it is important to note that there is no published work that specifically investigated the toxicity of QS, and thus, the mechanism behind QS toxicosis is yet to be explained. Given this general perception of oak toxicity and the results from supplementary experiments on QS extracts, it can be proposed that further exploration of this topic could be eminently beneficial.

## Technological applications of *Quercus salicina* Blume extracts

Apart from the nutritional aspect of herbs and natural food collected from trees, QS can contribute with great impact to food security and diversification of human diet (Vinha et al., 2016). Plant-derived polyphenols are generally used as antioxidants in food to increase shelf-life and to prevent oxidative deterioration. Many studies focused on the positive effects of antioxidants in food application regarding the prevention of chronic degenerative disease, cardiovascular diseases and cancer. Although dried leaves of QS were commonly utilized for extraction, previous works have proven that exploiting other parts of this plant such as the stem, bud and bark would equally be valuable based on total phenolic and flavonoid contents identified in them. Table 3 lists down the brief descriptions of past researches regarding the exploitation of the bioactive properties of QS through its application in different industry sectors such as medicine, food and cosmetics. Available studies on the potential application of QS as a functional ingredient in products are mostly related with medicine and cosmetics. Nonetheless, the increasing interest of consumers in all-natural products presents an opportunity for QS to be also utilized in producing functional and health-promoting food products apart from its current application as functional tea.

**Table 3:** Potential applications of *Quercus salicina* Blume in various industrial sectors

Industry sector	Product application	Function	Major findings	References
Pharmaceutical/cosmetic	Cosmetic products	Antioxidant, tyrosinase inhibitor and elastase inhibitor	With its antioxidant, anti-aging and whitening properties, the agylcone fraction of QS extracts could be utilized as a functional cosmetic ingredient	Lee and Park (2011)
	Topical cream	Antimicrobial, anti-inflammatory	At 0.25% concentration, ethyl acetate fraction of QS extracts was considered a natural preservative that can inhibit skin inflammation exacerbated by skin microflora (i.e. *Staphylococcus aureus*, *Bacillus subtilis*, *Propionibaterium acnes*, *Escherichia coli* and *Pseudomonas aeruginosa*)	Gu et al. (2014)
	Treatment for athlete’s foot	Antimicrobial	Extracts from QS leaves had antimicrobial activity against *Trichophyton mentagrophytes* wherein the inhibitory effect of QS extract was found to be better than that of cosmetic antifungal agents phenoxyethanol and methylparaben at 0.2–0.4% concentration	Jang et al. (2018)
Medicinal	Treatment for urolithiasis	Antioxidant	QS extracts suppressed the production of free radicals and subsequent lipid peroxidation in the kidneys, preventing renal tubular epithelial cell injury and calcium oxalate stone formation	Moriyama et al. (2009)
	Treatment for rheumatoid arthritis (analgesic)	Anti-inflammatory, antinociceptive	Quercetin isolated from the ethyl acetate fraction of QS extract was found to be an active component against oxidative stress in rheumatoid arthritis	Lee et al. (2012)
	Antidiabetic drug	Antioxidant	Hot water extracts of QS prevented the oxidation and eventual death of pancreatic β cells which could significantly affect the secretion of insulin	Song et al. (2013)
	Medicine for cardiovascular disease (endothelium-dependent vasodilator)	Vasorelaxant	Ethanolic extract of QS induced the phosphorylation of endothelial nitric oxide synthase and the subsequent activation of guanylyl cyclase which are known modulators of vascular function	Park et al. (2016)
Food	Beverage	Anti-lithiasis	This patent presented an alternative method of producing QS leaf extracts with higher retention of bioactive components. This method involved an enzyme inactivation step, and alcohol and water extraction steps	Han (2012)
	Beverage	Anti-lithiasis	This patent proposed an extracting method which claimed to solve the problems involved in previous patents regarding manufacture of QS extract	Yu et al. (2014)
	Food supplement	Antioxidant (functional food ingredient)	The application of superfine grinding on QS leaves through ball milling to produce functional powders increased their total phenolic and flavonoid contents; therefore, antioxidant activities of the superfine grounded powders increased	Hong (2019)

## Future perspectives

*Quercus salicina* Blume has been used as folk medicine for many years, and scientific findings strongly linked its medicinal properties with its anti-oxidative activities which were measured through various radical scavenging assays. A wide array of phenolic and flavonoid compounds in QS had been identified and the concentrations of these components would vary depending on the extracting medium and parts of the plant. Generally, researches which defined the characteristics of QS extracts, including their individual components and biological activities, are necessary to validate their feasible application as a novel food material. Current consumer trends in the food and nutraceutical industries further support the incorporation of QS extracts in food products since non-synthetic ingredients with high concentrations of bioactive compounds are strongly favored in developing product innovations.

Taking all these into consideration, further researches geared towards valorizing QS as a natural source of bioactive compounds or as health-promoting additives for new foodstuffs are highly recommended. Likewise, the mechanisms of toxicity in QS must also be further studied in order to be fully understood and to be able to establish non-toxic concentration levels for the purpose of registering it as a safe food ingredient.
