# Correction to: Bioactive compounds and antioxidant activities of *Quercus salicina* Blume extract

**DOI:** 10.1007/s10068-020-00787-7

**Published:** 2020-07-08

**Authors:** Thinzar Aung, Marie Anna Dominique Bibat, Chang-Cheng Zhao, Jong-Bang Eun

**Affiliations:** Department of Integrative Food, Bioscience and Biotechnology, Graduate School of Chonnam National University, Gwangju, 61186 South Korea

## Correction to: Food Sci Biotechnol (2020) 29(4):449–458 10.1007/s10068-020-00755-1

The article “Bioactive compounds and antioxidant activities of *Quercus salicina* Blume extract”, written by Thinzar Aung, Marie Anna Dominique Bibat, Chang-Cheng Zhao and Jong-Bang Eun, was originally published Online First without Open Access. After publication in volume 29, issue 4, pages 449–458 the author decided to opt for Open Choice and to make the article an Open Access publication. Therefore, the copyright of the article has been changed to © The Author(s) 2020 and the article is forthwith distributed under the terms of the Creative Commons Attribution 4.0 International License (http://creativecommons.org/licenses/by/4.0/), which permits use, duplication, adaptation, distribution and reproduction in any medium or format, as long as you give appropriate credit to the original author(s) and the source, provide a link to the Creative Commons license, and indicate if changes were made.

The original article has been corrected.

